# Corneal wound healing and nerve regeneration by novel ophthalmic formulations based on cross-linked sodium hyaluronate, taurine, vitamin B6, and vitamin B12

**DOI:** 10.3389/fphar.2023.1109291

**Published:** 2023-02-02

**Authors:** Claudio Bucolo, Grazia Maugeri, Salvatore Giunta, Velia D’Agata, Filippo Drago, Giovanni Luca Romano

**Affiliations:** ^1^ Department of Biomedical and Biotechnological Sciences, School of Medicine, University of Catania, Catania, Italy; ^2^ Center for Research in Ocular Pharmacology-CERFO, University of Catania, Catania, Italy

**Keywords:** corneal wound healing, nerve regeneration, vitamin B6, vitamin B12, taurine, sodium hyaluronate

## Abstract

**Introduction:** To evaluate the pharmacological profile of ocular formulations based on cross-linked sodium hyaluronate (CL-SH), taurine (Tau), vitamin B6 (Vit B6) and vitamin B12 (Vit B12) using *in vitro* and *in vivo* paradigms.

**Methods:** Rabbit corneal epithelial cells were used to assess wound healing and reactive oxygen species (ROS) formation by scratch assay and oxidative stress (0.3 mM H_2_O_2_; 30 min), respectively with or without ocular formulations exposure. *In vivo* studies were carried out on albino rabbits to evaluate corneal nerve regeneration and corneal wound healing with or without treatment with six different formulations. Animals were anesthetized, the corneal epithelium was removed, and formulations were topically administered (30 μL/eye; 3 times/day for 6 days). Slit-lamp observation was carried out at different time points. After 6 days the animals were killed, and corneas were collected to evaluate corneal re-innervation by immunohistochemistry of selective neuronal marker β-III tubulin.

**Results:** Formulations containing the concentrations 0.16% or 0.32% of cross-linked sodium hyaluronate, taurine, vitamin B6 and vitamin B12 accelerated corneal wound healing. Cells exposed to H_2_O_2_ led to significant (*p* < 0.05) increase of reactive oxygen species concentration that was significantly (*p* < 0.05) counteract by formulations containing cross-linked sodium hyaluronate (0.32%) and taurine with or without vitamins. The extent of re-innervation, in terms of β-III tubulin staining, was 5-fold greater (*p* < 0.01) in the eye of rabbits treated with formulation containing 0.32% cross-linked sodium hyaluronate, taurine, vitamins (RenerviX^®^) compared with the control group (no treatment). Furthermore, re-innervation elicited by RenerviX^®^ was significantly greater (*p* < 0.01) compared with the group treated with the formulation containing 0.32% cross-linked sodium hyaluronate and taurine without vitamins, and with the group treated with the formulation containing 0.5% linear sodium hyaluronate (SH), taurine, and vitamin B12, respectively.

**Discussion:** In conclusion, among the formulations tested, the new ophthalmic gel RenerviX^®^ was able to contrast oxidative stress, to accelerate corneal re-epithelization and to promote nerve regeneration.

## Introduction

Corneal damage represents a frequent clinical problem consequent to various chemical, physical, and pathological insults, including, but not limited to, dry eye disease and refractive surgery (Ljubimov and Saghizadeh, 2015; Bandeira et al., 2019)**,** that generate a potent inflammatory response (Mohan et al., 2022). Oxidative stress has been demonstrated to play a central role in ocular inflammation eliciting reactive oxygen species that contribute to ocular surface damage (Cejkova and Cejka, 2015). Based on these premises, antioxidants may represent a potential option to handle corneal damage (Dogru et al., 2018) elicited by inflammatory process (Buddi et al., 2002; Jurkunas et al., 2010; Shetty et al., 2017; Fresta et al., 2020). Corneal wound healing is a complex and dynamic process which helps to preserve the integrity of the corneal epithelial to ensure corneal transparency and clear vision. This process includes, above all, the migration, proliferation, adhesion, and differentiation of the stem cell of the corneoscleral junction, and the remodeling of extracellular matrix (Mei et al., 2012; Di Girolamo et al., 2015; Ljubimov and Saghizadeh, 2015; West et al., 2015; Chou et al., 2018), regulated by many cytokines, growth factors, and signaling pathways (Mohan et al., 2022). Furthermore, the preservation of corneal nerves is crucial for normal corneal function but also in promoting epithelial wound healing thanks to the release of essential neurotrophins for corneal homeostasis (Bucolo et al., 2009; Cortina et al., 2010; Bucolo et al., 2019; Puglia et al., 2021). Therefore, after a corneal damage, it is essential to restore the epithelium, the stroma, but also the nervous components (Bukowiecki et al., 2017; Wilson et al., 2017). As a result, corneal repair and regenerative strategies should target multiple pathways and mechanisms, and several approaches have been investigated to maintain corneal homeostasis and healing process. For example, the extracellular matrix components (such as proteoglycans) regulate collagen deposition and matrix assembly, and while sodium hyaluronate demonstrated to accelerate the healing of corneal epithelial after injury (Mohan et al., 2003; Borderie et al., 2006; Yang et al., 2010; Wu et al., 2013; Gupta et al., 2022). Moreover, vitamins have a role in promoting the healing after damage and in maintaining the normal cell growth, replication processes and reinnervation (Kim et al., 2012; Romano et al., 2014; Reins et al., 2016; Fernandez-Villa et al., 2018; Fogagnolo et al., 2020; Gujral et al., 2020). The research of topical products able to modulate the wound healing is growing fast to find new approaches to handle corneal damage. This study aims to evaluate the pharmacological profile of different ocular formulations based on sodium hyaluronate (linear and cross-linked) at different concentrations, taurine, vitamin B6 and vitamin B12 using *in vitro* and *in vivo* paradigms.

## Material and methods

### Cell culture

Statens Seruminstitut rabbit corneal (SIRC) epithelial cells (ATCC CCL-60) were cultured in Eagle’s Minimum Essential Medium (EMEM, Sigma-Aldrich, Milan, Italy) supplemented with 10% of fetal bovine serum (FBS, Sigma-Aldrich), 1X Minimum Essential Medium Non-Essential Amino Acids (MEM NEAA, Thermo Fisher Scientific, Waltham, MA, United States) and 1X Penicillin/Streptomycin (P/S, Sigma-Aldrich) at 37 °C in 5% CO_2_ in humid air. Cell culture plates were coated with 5–10 µL gelatin solution/cm^2^ (i.e., 0.1–0.2 mg/cm^2^ gelatin, G1393, Sigma-Aldrich) to promote cell adhesion. SIRCs (P6) were cultured with or without test formulations. All media were filtered with syringe filters, 0.45 µm (Corning^®^ 28 mm Diameter Pore SFCA Membrane, Cat. No. 431220, Arizona, United States) to ensure sterile conditions.

### Ophthalmic formulations

Six different ophthalmic formulations were used: formulation #1 (F1), containing 0.5% SH-L, Tau and 0.05% Vit B12; formulation #2 (F2), containing 0.48% cross-linked SH-CL, 0.5% Tau, 0.05% Vit B6 and 0.05% Vit B12; formulation #3 (F3), containing 0.32% SH-CL, 0.5% Tau, Vit B6 and Vit B12 (Renervix^®^ Alfa Intes I.T.S. s.r.l); formulation #4 (F4), containing 0.16% SH-CL, 0.5% Tau, Vit B6 and Vit B12; formulation #5 (F5), containing 0.02% SH-CL, 0.5% Tau, Vit B6 and Vit B12; formulation #6 (F6), containing 0.32% SH-CL, 0.5% Tau (no vitamins).

### Scratch wound healing assay

The SIRC cells were grown to confluence in six-well dishes (5 × 10^4^ cells/well). Reached the confluence, cells were washed twice with warm phosphate saline buffer (PBS, 1X) and then incubated with a serum-free medium for 5 h. Then, the confluent monolayer of cells was scratched with a 200 μL pipette tip. All the wells were washed with fresh medium to remove detached cells before incubation in a serum-free medium containing formulation #1, formulation #2, formulation #3, formulation #4, formulation #5 or formulation #6. To be sure the wounds with the same wound area were compared, a couple of lines were made at two points of the well to link the opposite points in the well with a marking pen, using the lines as a reference for the photographic report at the time of the beginning of the experiment (T0) and for 12 h (T12), 24 h (T24), 36 h (T36), 48 h (T48) and 72 h (T72). Wound area was analyzed from six different wells for each treatment, and all images were acquired with a Leica microscope using a ×20 magnification. The average wound area, expressed in the percentage of control (CTR), was determined using ImageJ Software (Broken Symmetry Software, Bethesda, MD, United States).

### Detection of ROS

ROS generation was evaluated in SIRC cells, after oxidative stress induction by treatment with H_2_O_2_, by using the 2′,7′–dichlorofluorescin diacetate (DCFDA)– Cellular Reactive Oxygen Species Detection Assay Kit (ab113851, Abcam, Cambridge, United Kingdom) according to the manufacturer’s protocol, as previously described by Maugeri et al. (Antioxidants 2022. PMID: 35052632). Briefly, SIRC cells were plated into 96-well plates (1 × 10^4^ cells/well). After overnight growth, cells were cultured for 60 min in the control medium (CTR); or in the presence of the formulation #3, containing 0.32% sodium hyaluronate (SH-CL), 0.5% taurine, 0.05% vitamin B6 and 0.05% vitamin B12 (RenerviX^®^); or in the presence of formulation #6, containing 0.32% sodium hyaluronate (SH-CL) and 0.5% taurine. Then, oxidative stress was induced with 0.3 mM H_2_O_2_ treatment for 30 min. Subsequently, cells were washed gently in PBS twice and incubated with 25 μM DCFDA previously dissolved in a buffer solution for 45 min in the dark. ROS concentration was detected by fluorescence spectroscopy with excitation and emission wavelength of 495 nm and 529 nm, respectively, using Varioskan Flash Multimode Reader (Thermo Fisher Scientific). Twelve replicate wells were used for each group.

### Corneal epithelial wound healing

Male New Zealand albino rabbits (1.8–2.0 kg) were purchased from Envigo (Udine, Italy). Animals were housed under standard conditions with food and water provided *ad libitum* in a light-controlled room and set temperature and humidity. Animal care and experimental procedures were carried out according to the ARVO Statement for the Use of Animals in Ophthalmic and Vision Research. Protocols were approved by the Institutional Animal Care and Use Committee of the University of Catania (project #303). Animals were anesthetized and the corneal epithelium was removed with 0.5 mm corneal rust ring remover (Algerbrush, EyeBM Vet, Milan, Italy), under a dissecting microscope. The eyes were treated as follow: group 1) Formulation #6 containing 0.32% SH-CL and 0.5% Tau; group 2) Formulation #3 containing 0.32% SH-CL, 0.5% Tau, 0.05% Vit B6 and 0.05% Vit B12, RenerviX^®^); group 3) Formulation #1 containing 0.5% SH-L, 0.5% Tau and 0.5% Vit B12. All formulations were topically administered (one drop, three times per day for 6 days) starting the same day of corneal epithelial debridement. Slit-lamp observation was carried out at different time points. After 6 days, the animals were killed, and corneas were collected for the immunohistochemical analysis to evaluate corneal re-innervation by immunohistochemical analysis of the selective neuronal marker beta-III tubulin.

### Immunohistochemistry analysis

The expression and distribution of ß-III tubulin in rabbit cornea were evaluated through immunohistochemical analysis. Briefly, after dewaxing in xylene, the corneal slides were hydrated through graded ethanol and incubated for 30 min in 0.3% H_2_O_2_/methanol to quench endogenous peroxidase activity and then rinsed for 20 min with phosphate-buffered saline. The sections were then heated in a thermoregulated bath (80° for 30 min) with rodent decloaker (Biocare Medical, Pacheco, CA, United States), to perform antigen retrieval. The blocking step to prevent non-specific binding of the antibody was performed before application of the primary antibody with 1% bovine serum albumin (BSA, Sigma, Milan, Italy) in PBS for 1 h in a moist chamber. After blocking, the sections were incubated overnight at 4 °C with ß-III Tubulin antibody (ab78078, Abcam, Cambridge United Kingdom), work dilution in PBS and 1%BSA 1:100. Immune complexes were then treated with a biotinylated link antibody (HRP-conjugated anti-rabbit was used as secondary antibodies) and then detected with peroxidase labeled streptavin, both incubated for 10 min at room temperature (LSAB + System-HRP, K0690, Dako, Denmark). The immunoreaction was visualized by incubating the sections for 3 min in 3,3′-diaminobenzidine solution (DAB substrate Kit; SK-4100, Vector Laboratories, Burlingame, CA, United States). The samples were lightly counterstained with hematoxylin, mounted in vecta mount (Vector Laboratories) and observed with an Axioplan Zeiss light microscope (Carl Zeiss) and photographed with a digital camera (AxioCam MRc5, Carl Zeiss). Densitometric analysis was carried with ImageJ. ß-III Tubulin staining in the corneal epithelium was quantified as previously described by Romano et al. (2014).

### Statistical analysis

Statistical analysis was performed by GraphPad prism 7 (GraphPad software La Jolla, California). The data generated by all experiments are reported as mean ± SD. One-way analysis of variance (ANOVA) was carried out, and Tukey's *post hoc* test was used for multiple comparisons. Differences between groups were considered statistically significant for *p*-values <0.05.

## Results

### Wound healing in SIRCs

**FIGURE 1:**
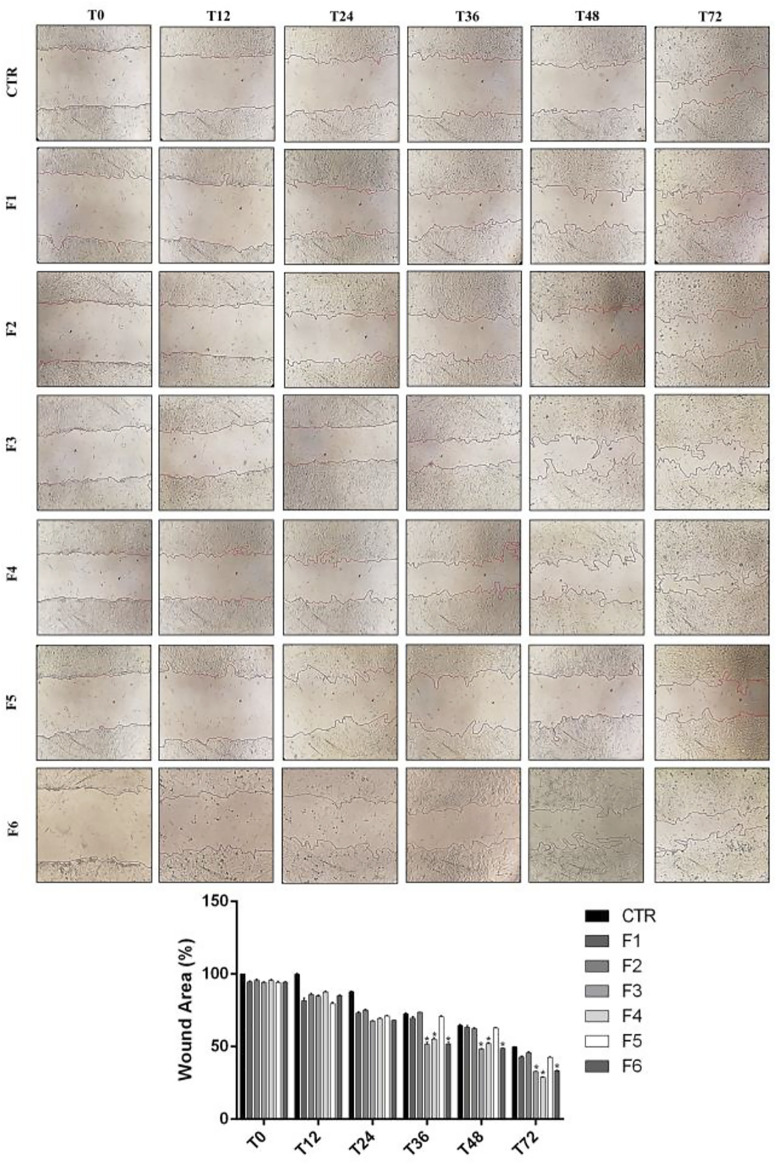
Wound healing in SIRCs monolayer. (Top Panel) Representative images of wound healing assays performed in SIRCs exposed to the six different formulations at 0, 12, 24, 36, 48 and 72 h. (Bottom Panel) The bar graph shows the average wound area expressed in the percentage of CTR. *p < 0.01 vs. F1, F2 and F5 as determined by one-way ANOVA followed by the Tukey's *post hoc* test. Data are shown as mean ± SD of five independent experiments (*n* = 5).

**FIGURE 2:**
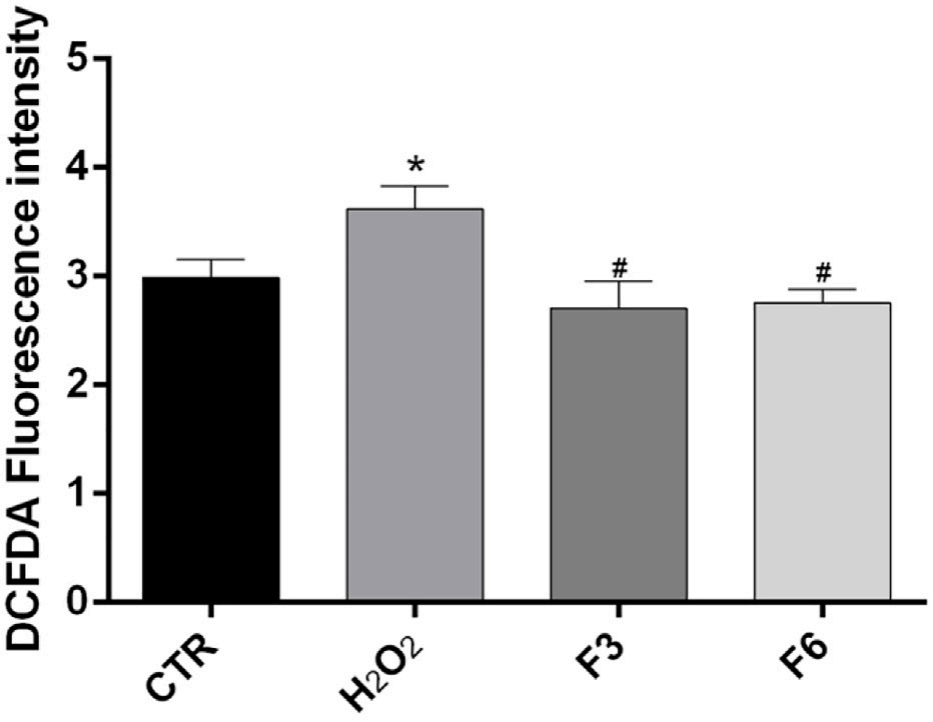
Detection of intracellular ROS production in corneal cells.

We performed wound healing assay to evaluate the impact of the formulations in the wound repair capability of SIRC cells. As shown in Figure 1, at 12 and 24 h after confluent SIRCs were scratched, all formulations produced a significant (*p* < 0.01) reduction of the average wound area as compared to control. However, starting from 36 h until to 72 h, the corneal cells exposed to F3, F4, and F6 showed the best performance in terms of wound closure compared to control group and the other formulations (*p* < 0.01 vs. F1, F2, F5). No significant differences were observed between F3, F4, and F6. These findings suggest that these formulations exert comparable positive effects on the wound healing rate in SIRC cells. We then analyzed the effect of F3 against oxidative stress induced by treatment with H_2_O_2_ (0.3 mM) for 30 min using a DCFDA assay. To assess the role of vitamins contained in F3, we also tested F6, containing similarly to F3, 0.32% SH-CL and 0.5% Tau, but no vitamins. As shown in Figure 2, cellular ROS levels significantly (*p* < 0.05) increased in SIRC cells after H_2_O_2_ treatment compared to control. The treatment with F3 and F6 significantly (*p* < 0.05) reduced ROS formation after H_2_O_2_ stress.

ROS levels were measured in SIRCs after 0.3 mM H_2_O_2_ treatment for 30 min alone or in cells previously treated for 60 min with F3 or F6, using the cytoplasmic probe, DCFDA. *p < 0.05 vs. CTR; #*p* < 0.05 vs. H_2_O_2_ as determined by one-way ANOVA followed by Türkiye’s multiple comparison test. Data are shown as mean ± SD of five independent experiments (*n* = 5).

### Corneal epithelial wound healing *in vivo* study

**FIGURE 3:**
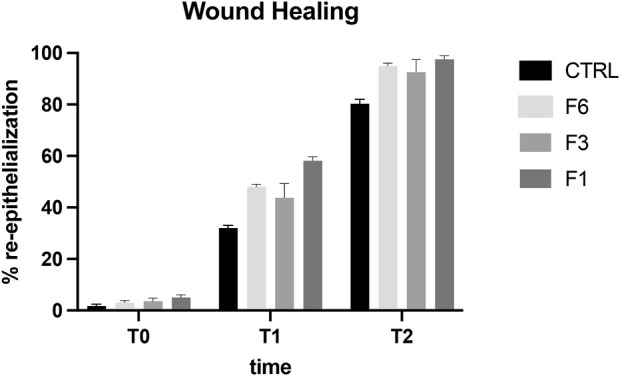
Wound healing in rabbit eye. T0 (day 1), T1 (day 2), T2 (day 4). Formulation 6 (F6); RenerviX^®^(F3); Formulation 1 (F1); Control group. Data are showed as % of corneal re-epithelialization. No statistical differences were observed between treated groups. Data are shown as mean ± SD (*n* = 4).

The aim of the *in vivo* study was to evaluate the effects of RenerviX^®^ on corneal wound healing and to evaluate the expression and the localization of regenerated nerve fibers after corneal abrasion in rabbit eye. As showed in Figure 3 all the ophthalmic formulations tested [Formulation 6 (F6) containing 0.32% SH-CL and 0.5% Tau; Formulation 3 (F3) (RenerviX^®^) containing 0.32% SH-CL, 0.5% Tau, 0.05% Vit B6 and 0.05% Vit B12; Formulation 1 (F1) containing 0.5% SH-L, 0.5% Tau and 0.5% Vit B12] contribute to the corneal wound healing even though no statistical differences were observed between treated groups (Figure 3).

### Effects of ocular formulations on corneal nerve regeneration

**FIGURE 4:**
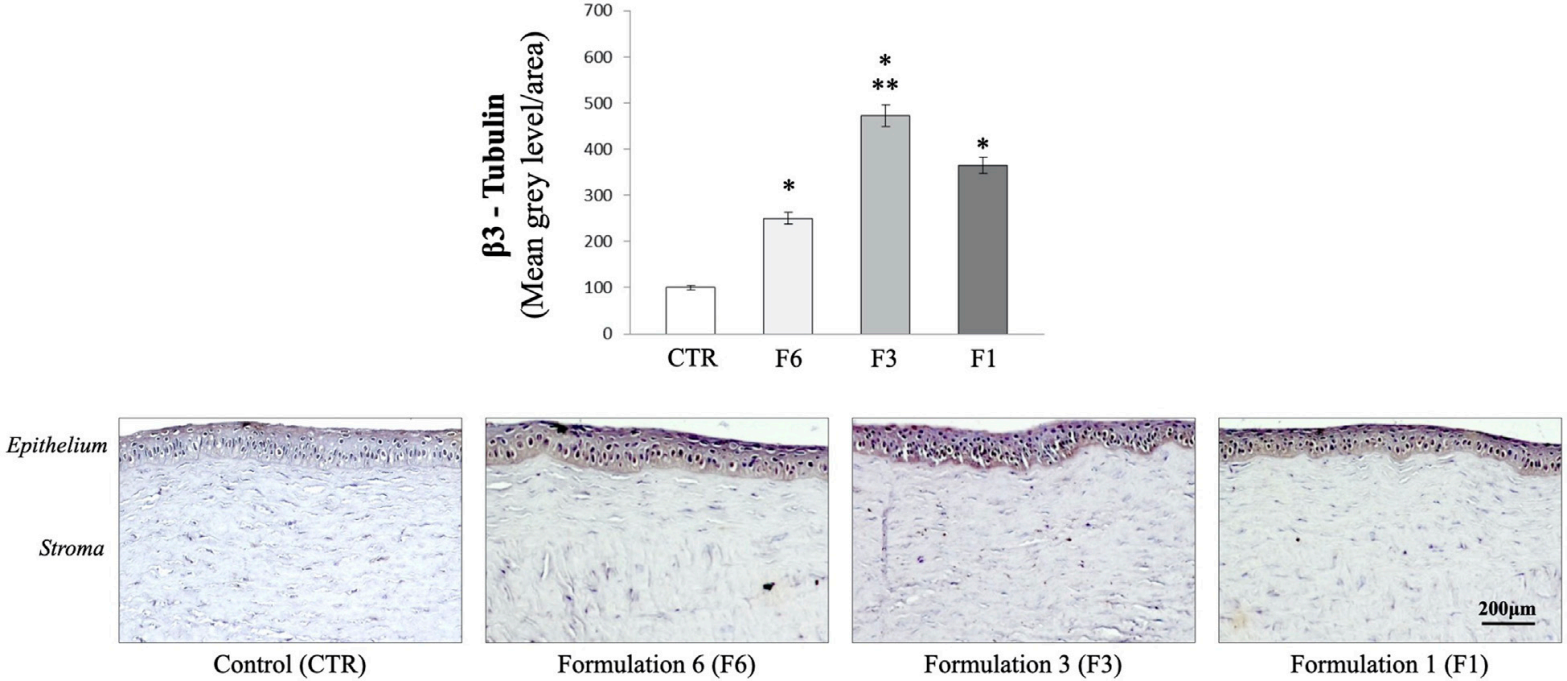
Immunohistochemical analysis. Measurement of corneal ß-III tubulin expression. Formulation 6 (F6); Formulation 3 (F3, RenerviX^®^); Formulation 1 (F1); Control group. *p < 0.01 vs. CTR; **p < 0.01 vs. F6 and F1. Data are shown as mean ± SD (*n* = 4).

Corneal re-innervation was examined by immunohistochemical analysis of the selective neuronal marker, beta-III tubulin after mechanical injury. As shown in Figure 4, immunohistochemical analysis demonstrated the presence of regenerating nerve fibers expressing β-III tubulin in the apical areas of the cornea of eyes treated with all three formulations (F1; F3 and F6) (*p* < 0.05 and *p* < 0.01 vs. CTR). However, the extent of re-innervation was significantly greater in the eye of rabbits treated with RenerviX^®^ compared with the group control and the groups treated with formulations containing 0.32% SH-CL and Tau (F6) and formulation containing 0.5% SH-L, 0.5% taurine and 0.5% vitamin B12 (F1) (*p* < 0.05 vs. F6 and F1).

## Discussion

In the present study we demonstrated that RenerviX^®^, was able to improve corneal wound healing, to restore functional corneal nerves, and to protect corneal cells from oxidative stress. Treatment with RenerviX^®^ stimulates re-innervation of the injured cornea in rabbit eye with a significant difference when compared to formulation 6 and formulation 1. Finally, no levels of taurine, pyridoxine (vit B6) and cyanocobalamin (vit B12) were detected after 18 h in the cornea samples of rabbit eyes treated with a single instillation of RenerviX^®^, suggesting that no deposit of these molecules occurred after topical administration. Altogether, these findings suggest that pyridoxine (vitamin B6) present in RenerviX^®^, significantly contributes to the corneal preservation and recovery after an insult.

Previous studies demonstrated the importance of vitamins in maintaining ocular surface homeostasis, suggesting the possible protective effects against damages (Lasagni Vitar et al., 2022). Vitamins are essential for many corneal functions and help ensuring corneal integrity supporting the epithelial barrier and cells survival (Yin et al., 2011; Bucolo et al., 2015; Reins et al., 2015; Gozzo et al., 2021; Kaminska et al., 2021; Lasagni Vitar et al., 2022). Moreover, their anti-inflammatory, antimicrobial and antioxidant properties have been demonstrated (Wimalawansa, 2019). Vit B6 role is important for several biosynthetic pathways such as purine, pyrimidine, and amino acids syntheses and in maintaining the normal cell growth and replication processes (Fernandez-Villa et al., 2018; Fiorillo and Romano, 2020; Fogagnolo et al., 2020). For example, the local treatment with Vit B12 led to faster repair of corneal damage and facilitated reinnervation (Romano et al., 2014). This is in line with the evidence that Vit B12 promotes the synthesis of neurotrophic factors, supporting neurite growth and survival (Scalabrino and Peracchi, 2006; Okada et al., 2010). Indeed, Vit B12 deficiency is associated with sensory innervation impairment, optic neuropathy, eye movement disorders and corneal damage (Chavala et al., 2005; Akdal et al., 2007; Jurkunas et al., 2011; Conti et al., 2021).

Moreover, Vit B6 has been recognized as a potent antioxidant as well as an established cofactor for several metabolic enzymes, including, among others, those involved in protein metabolism, conversion of tryptophan to niacin, and neurotransmitter function (Kannan and Jain, 2004; Tunali, 2014; Hsu et al., 2015).

The role of Vit B6 as a therapeutic agent has been demonstrated in several disorders such as diabetes (Jain, 2007; Amato et al., 2021) and cardiovascular diseases (Wierzbicki, 2007). For example, the antioxidant and scavenging properties have been considered in reducing oxidative stress markers associated with homocysteinemia or in preventing free radicals formation and lipid peroxidation in cellular models (Mahfouz and Kummerow, 2004).

In addition, Vit B6 is involved in the immune system regulation and the regulation of neurotransmitters (Baltrusch, 2021). Being essential for the amino-acid metabolism, Vit B6 regulates the synthesis of neurotransmitters, responsible for signal transmission (Yang and Wang, 2009; Baltrusch, 2021). In preclinical models, vitamin B6 showed neuroprotective effects against glutamate damage stimulating nerve regeneration, and prevention of neuronal death in the retina after ischemic damage (Wang et al., 2002; Yang and Wang, 2009). Furthermore, some clinical evidence supported the regenerative effect of Vit B6 (Talebi et al., 2013). These evidences are important with an impact on corneal nerves protection necessary for the maintenance of a healthy ocular surface (Muller et al., 2003) and support corneal healing (Yu and Rosenblatt, 2007; He et al., 2010; Marfurt et al., 2010; Toro et al., 2021).

In conclusion, among the formulations tested, the new ophthalmic gel based on 0.32% SH-CL, 0.5% taurine, 0.05% vitamin B6 and 0.05% Vitamin B12 (RenerviX^®^), demonstrated to better contrast oxidative stress, to accelerate corneal re-epithelization and to promote nerve regeneration, suggesting an important advantage in clinical practice, ranging from corneal abrasion and/or neuropathy (diabetes or severe dry-eye) to help patients’ recovery after eye surgery.

## Data Availability

The raw data supporting the conclusion of this article will be made available by the authors, without undue reservation.
